# Personalized Feedback from Wearables to Enhance Health Behaviour, Adherence, and Outcomes in Aging and Dementia Research

**DOI:** 10.1002/alz70858_105206

**Published:** 2025-12-26

**Authors:** Angela C. Roberts, Emily J Rogalski, SuperAging Research Initiative, Ontario Neurodegenerative Disease Research Initiative

**Affiliations:** ^1^ Western Institute for Neuroscience, Western University, London, ON, Canada; ^2^ Canadian Centre for Activity and Aging, London, ON, Canada; ^3^ Western University, London, ON, Canada; ^4^ Healthy Aging & Alzheimer's Research Care (HAARC) Center, University of Chicago, Chicago, IL, USA; ^5^ University of Chicago, Chicago, IL, USA

## Abstract

Wearable technologies provide continuous, real‐world data on mobility, cognition, and health behaviours, creating new opportunities to support aging individuals, including those with mild cognitive impairment (MCI), Alzheimer's disease, and related dementias (ADRD). Translating insights derived from wearables into meaningful, personalized feedback can foster sustained behaviour change and enhance adherence in clinical trials and observational studies. Through a multidisciplinary lens, this session will illustrate the challenges and opportunities associated with delivering personalized feedback from wearables, specifically focusing on the following topics: 1) co‐design of feedback mechanisms from wearables to improve adherence and study retention in dementia and aging research, 2) strategies for integrating multimodal sensor data into personalized interventions that drive meaningful behaviour change, and 3) lessons learned from real‐world implementations in clinical trials and longitudinal studies.

Lessons drawn from wearable‐based clinical trials and observational studies in aging and ADRDs, including the Ontario Neurodegenerative Disease Research Initiative, the Communication Bridge Trials and the SuperAging Research Initiative, illustrate effective co‐design processes for delivering personalized feedback to individuals with cognitive impairment. Recent findings from the Health in Aging, Neurodegenerative Diseases, and Dementias in Ontario study demonstrate the types and mechanisms of health behaviour changes resulting from wearable‐sensor‐derived personalized feedback. These studies highlight how tailored feedback can influence health behaviour changes, retention, and adherence. Challenges faced when generating and delivering personalized feedback include ensuring it remains valuable and understandable for individuals with cognitive difficulties, balancing comprehensive data collection with participant‐friendly feedback approaches, and providing sufficient data fidelity for reliable and valid feedback. This session will outline practical strategies for overcoming these challenges, including evidence‐based behavioural feedback models, best practices for co‐designing interventions with patients, care partners, and clinicians, incorporating outcomes with high interpretability, and transparent analytics pipelines featuring sensor interoperability.

By integrating personalized feedback from wearable sensors into dementia and aging research, we can enhance health behaviours, improve participant retention, and maximize the effectiveness of clinical trials and observational studies. This session will offer a scientific and practical framework for utilizing wearable technology to foster engagement, facilitate behaviour change, and enhance health outcomes in older adults and those with ADRD.

